# Emergent many-body composite excitations of interacting spin-1/2 trimers

**DOI:** 10.1038/s41467-022-34342-1

**Published:** 2022-11-12

**Authors:** Anup Kumar Bera, S. M. Yusuf, Sudip Kumar Saha, Manoranjan Kumar, David Voneshen, Yurii Skourski, Sergei A. Zvyagin

**Affiliations:** 1 Solid State Physics Division, Bhabha Atomic Research Centre, Mumbai, 40085 India; 2 Homi Bhabha National Institute, Anushaktinagar, Mumbai, 400094 India; 3 S. N. Bose National Centre for Basic Sciences, Block JD, Sector III, Salt Lake, Kolkata, 700106 India; 4 ISIS Facility, STFC Rutherford Appleton Laboratory, Harwell Oxford, Didcot, OX11 0QX UK; 5 Department of Physics, Royal Holloway University of London, Egham, TW20 0EX UK; 6 Dresden High Magnetic Field Laboratory (HLD-EMFL), Helmholtz-Zentrum Dresden-Rossendorf, 01328 Dresden, Germany

**Keywords:** Magnetic properties and materials, Electronic properties and materials

## Abstract

Understanding exotic forms of magnetism in quantum spin systems is an emergent topic of modern condensed matter physics. Quantum dynamics can be described by particle-like carriers of information, known-as quasiparticles that appear from the collective behaviour of the underlying system. Spinon excitations, governing the excitations of quantum spin-systems, have been accurately calculated and precisely verified experimentally for the antiferromagnetic chain model. However, identification and characterization of novel quasiparticles emerging from the topological excitations of the spin system having periodic exchange interactions are yet to be obtained. Here, we report the identification of emergent composite excitations of the novel quasiparticles doublons and quartons in spin-1/2 trimer-chain antiferromagnet Na_2_Cu_3_Ge_4_O_12_ (having periodic intrachain exchange interactions *J*_1_-*J*_1_-*J*_2_) and its topologically protected quantum 1/3 magnetization-plateau state. The characteristic energies, dispersion relations, and dynamical structure factor of neutron scattering as well as macroscopic quantum 1/3 magnetization-plateau state are in good agreement with the state-of-the-art dynamical density matrix renormalization group calculations.

## Abstract (web summary)

It was recently predicted that, in addition to well-known spinon excitations, a 1D spin-trimer chain with periodic exchange couplings hosts novel composite excitations. Bera et al. experimentally demonstrate and characterize such excitations, termed doublons and quartons, in a spin-trimer compound Na_2_Cu_3_Ge_4_O_12_.

## Introduction

The magnetism in one-dimension (1D), dominated by quantum fluctuations, remains one of the extraordinary fundamental topics over the last century and has been explored extensively since the early days of quantum mechanics. In 1931, Bethe stated an ansatz to predict the ground state of 1D spin-1/2 Heisenberg antiferromagnetic chain (HAC) system^1^, and further extensions of this ansatz^2–5^ successfully predict the ground state as well as the excitations of the many-body interactions of the generalized 1D anisotropic Heisenberg–Ising (or XXZ) antiferromagnetic (AFM) model. Important predictions, such as the spinon excitations with fractional spin-1/2 quantum number, characteristic spinon continuum, as well as many body Bethe string states have subsequently been accurately calculated and precisely verified experimentally^6–11^. However, the utility of the Bethe ansatz is limited when perturbing the HAC beyond the solvable uniform XXZ model^12,13^, such as, with the periodic exchange interactions within the chain. One of such models is a quantum trimer spin-chain with repeating couplings *J*_1_-*J*_1_-*J*_2_ (intratrimer *J*_1_, and intertrimer *J*_2_)^14–19^ for which emergent composite excitations of novel quasi-particles doublons and quartons, in addition to the low energy fractional spinon excitations, have been predicted very recently^20^. Such emergent composite excitations appear only over a specific range of small values of *α* = *J*_2_/*J*_1_ < 0.4, and with increasing *α* value those states fractionalize to form the conventional spinon continuum when *α* → 1. However, a real material which meets these stringent requirements to support these composite excitations has thus far remained elusive. Here, we perform inelastic neutron scattering (INS) measurements on Na_2_Cu_3_Ge_4_O_12_ and report the experimental realization of the emergent composite excitations of the novel quasi-particles, doublons and quartons. The INS spectra reveal that the doublon and quarton excitations govern the high-energy dynamics, whereas spinon excitations are dominant at low energies. Our experimentally obtained eigenstates, dispersion relations, and dynamical structure factor are compared in detail to the state-of-the-art dynamical density matrix renormalization group (DMRG) calculations of the 1D spin-1/2 trimer model. The excellent agreement between the experiment and theory allows us the unambiguous identification and full characterization of the emergent composite excitations. Such emergent states are characterized by strong quantum entanglement, having promising prospective for quantum information processing^21^.

## Results

### Spin Hamiltonian and magnetization plateau

**Fig. 1:**
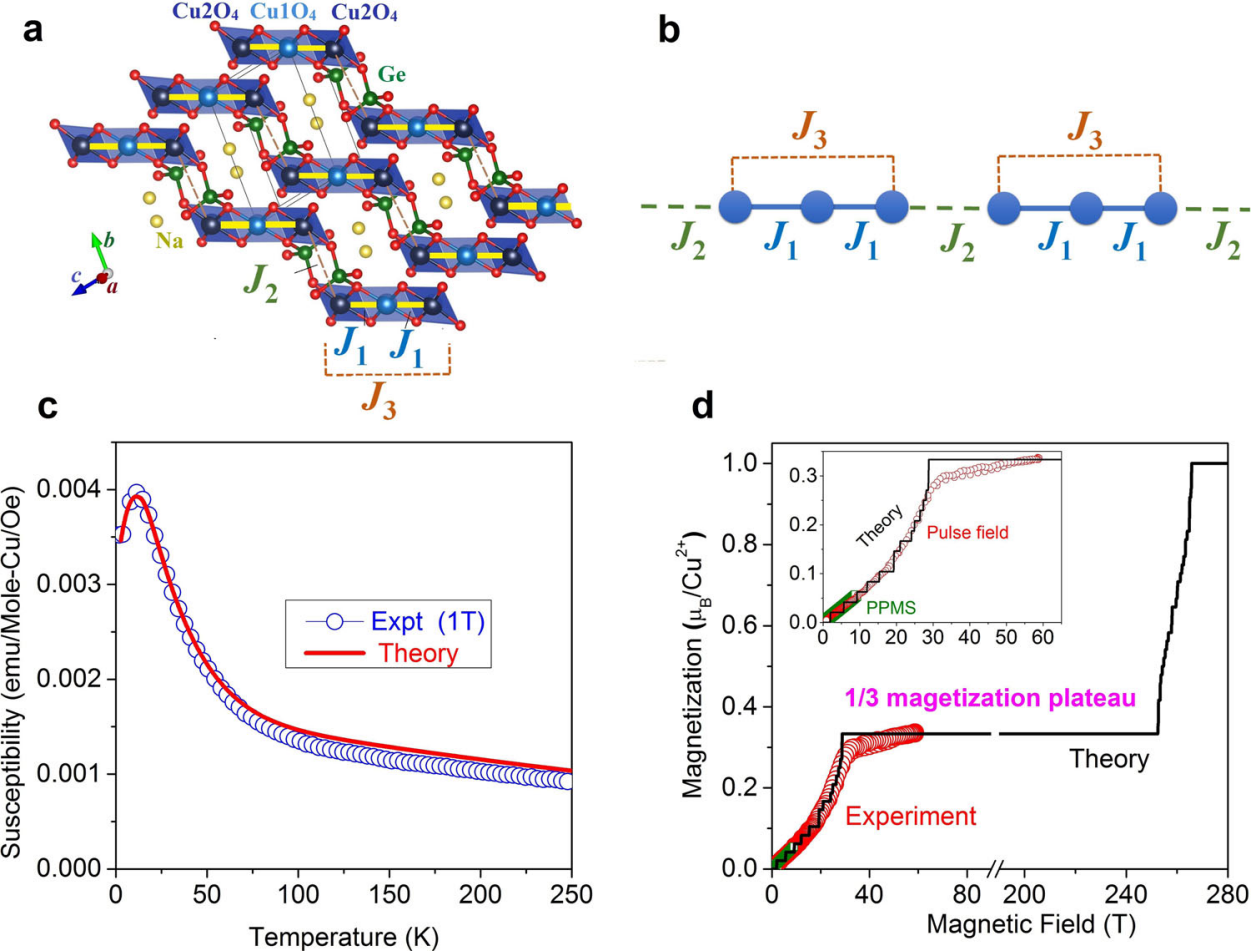
Crystal structure, magnetic model, magnetic susceptibility, and field-dependent magnetization of Na_2_Cu_3_Ge_4_O_12_. **a** The schematic spin-trimer structure of Na_2_Cu_3_Ge_4_O_12_. The spins S1, S2, and S3 are the three Cu^2+^ spins within a trimer unit. The *J*_1_, *J*_2_, and *J*_3_ denote the intratrimer, intertrimer and next-nearest neighbour intratrimer exchange couplings, respectively. **b** The schematics of 1D spin-chain with *J*_1_, *J*_2_ (=*αJ*_1_), and *J*_3_ (=*βJ*_1_). **c** The temperature-dependent susceptibility (*χ v*s.*T*) curve (open circles) of Na_2_Cu_3_Ge_4_O_12_ measured under a magnetic field (*μ*_0_*H*) = 1 Tesla. The calculated susceptibility curve as per the Hamiltonian (Eq. 1) with the parameters *J*_1_ = 235 K, *α* = 0.18, and *β* = 0.18 is shown by the red solid line. The calculations were performed by a hybrid ED/DMRG method up to *N* = 96. **d** The experimental isothermal magnetization (*M*) curve as a function of field *H* (red circles) of Na_2_Cu_3_Ge_4_O_12_, measured at 3.0 K by static field up to 9 T [using a Physical Property Measurement System (PPMS)] and the pulse field up to 59 T (at HLD, Dresden), respectively. The magnetization plateau *M/M*_*s*_ = 1/3 μ_B_/Cu^2+^ above 28 Tesla is found. The calculated *M*(*H*) curves of the model Hamiltonian in Eq. 1 with the parameters *J*_1_ = 235 K, *α* = 0.18, and *β* = 0.18 for *N* = 96 at *T* = 0 is shown by the black line. The calculation indicates that the plateau state persists up to a magnetic field of 252.5 T. The small steps in the calculation at low field are artefacts arising from the finite size of the cluster.

Na_2_Cu_3_Ge_4_O_12_ is an excellent realization of the paradigmatic spin model of spin-1/2 HAC comprising of coupled spin-trimers^22,23^. The crystal structure of Na_2_Cu_3_Ge_4_O_12_ is composed of Cu_3_O_8_ trimers formed by three edge-sharing CuO_4_ square planes in a linear fashion [Fig. 1a, b, and Supplementary Note 1]. The magnetic Cu^2+^ ions within the CuO_4_ square planes have quantum spin-1/2 (*S* = 1/2) providing a close approximation to the spin Hamiltonian

1 $$H=\mathop{\sum }\limits_{r=1}^{N/3}\left[{J}_{1}\left({\vec{S}}_{r,1}.{\vec{S}}_{r,2}+{\vec{S}}_{r,2}.{\vec{S}}_{r,3}\right)+{J}_{2}\left({\vec{S}}_{r,3}.{\vec{S}}_{r+1,1}\right)+{J}_{3}\left({\vec{S}}_{r,1}.{\vec{S}}_{r,3}\right)\right]$$

where ${\vec{S}}_{r,i}$ is a spin-1/2 operator at *i*^th^ (=1, 2, and 3) site of *r*^th^ spin-trimer. The positive *J*s denote the dominating antiferromagnetic exchange interactions with the nearest-neighbour (NN) superexchange interaction *J*_1_ within a given trimer via intermediated oxygen ions, intertrimer super-superexchange interaction *J*_2_ (=*αJ*_1_) via oxygen, germanium, and oxygen ions. The *J*_3_ (=*βJ*_1_) denotes the next-nearest-neighbour (NNN) intratrimer super-superexchange interaction between two edge spins of a given trimer [Fig. 1b] via oxygen-oxygen ions. For *α* = 0, the trimers are isolated from each other, while for *α* = 1 and *J*_3_ = 0 the model reduces to the isotropic Bethe-ansatz soluble HAC^1^. The antiferromagnetic interaction *J*_3_ competes with the interaction *J*_1_ and introduces a frustration in the spin system. In zero field, Na_2_Cu_3_Ge_4_O_12_ undergoes a magnetic phase transition to a long-range Néel type antifferomagnetic ordered state below the *T*_N_ ~ 2 K^22^ due to a weak interchain interaction *J*_4_ (=*γJ*_1_) [not included in Eq. (1)], through the super-super exchange pathways via the nonmagnetic Na^+^ and Ge^4+^ ions. Such weak interaction *J*_4_ is a small perturbation to the model Hamiltonian in Eq. (1) and slightly modifies the low energy excitations (discussed later). Nevertheless, above the *T*_N_, Na_2_Cu_3_Ge_4_O_12_ retains the main characteristics of the HAC comprising of coupled spin-trimers and well approximated by the 1D HAC model [Eq. (1)]. The experimentally measured temperature-dependent susceptibility [*χ*(*T*)] and the pulse-field magnetization [*M*(*H*)] curves are well reproduced by the model Hamiltonian in Eq. (1) [Fig. 1c, d] with the parameters *J*_1_ = 235 K, *α* = 0.18, and *β* = 0.18 (for details see ‘Methods’ and supplementary Fig. 6) and Landé *g*-factor *g* = 2.06 (as determined by ESR study; see Supplementary Note 3 for details). The presence of a 1/3 magnetization plateau, a characteristic feature of the weakly-coupled trimer spin systems, is found above *μ*_0_*H*_C1_ = 28 Tesla (T) both experimentally and theoretically [Fig. 1d]. Here, the 1/3-magnetization plateau is a macroscopic phenomenon of quantum origin. The plateau state is driven by the tendency of neighbouring antiferromagnetically coupled spins to form highly entangled spin-singlet states, leading to the spontaneously broken translational symmetry^15,24,25^. This plateau follows the Oshikawa-Yamanaka-Affleck rule^24^ *S.p* (1−*M*/*M*_sat_) = *Z* where *S* is the spin of the system, *p* is the number of spins per unit cell, *M* is the magnetization measured in the unit of saturation magnetization *M*_sat_, and *Z* is a set of integer numbers. Our precise DMRG calculations (system sizes up to *N* = 96) reveal that the 1/3 magnetization plateau state persists up to a magnetic field of *μ*_0_*H*_C2 _= 252.5 T and the field polarized state appears above *μ*_0_*H*_S_ ~ 265.8 T. The topologically nontrivial 1/3 magnetization plateau state can be characterized by a nonzero integer Chern number and appears due to the existence of finite gaps in the thermodynamic limit with the width of plateaus proportional to the gap size^17^. The topological origin of such phenomena of quantized magnetization plateaus relates the plateau state to a correlated topological insulator^17^.

### Energy level spectrum

**Fig. 2:**
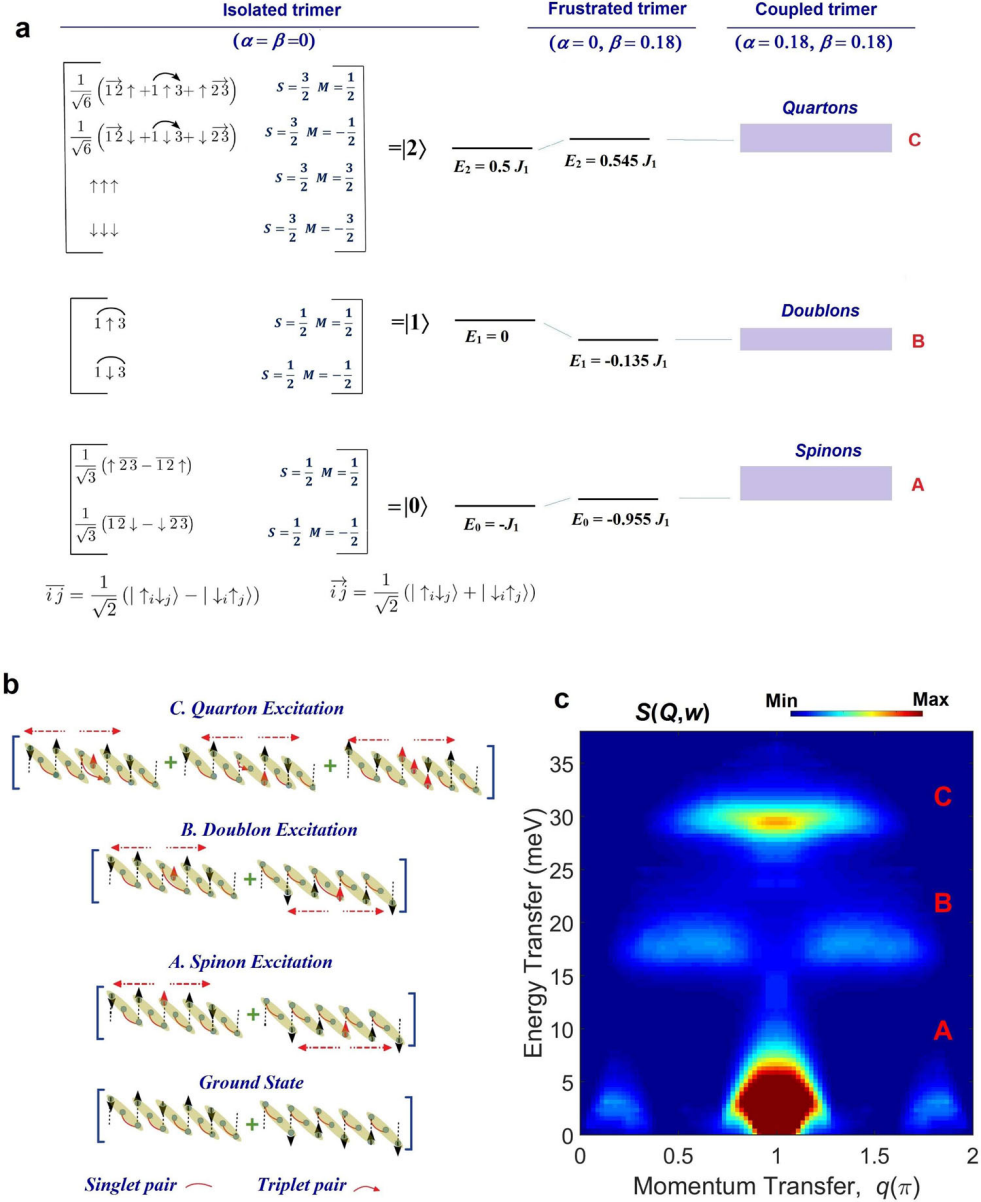
The energy level spectrum, wave functions, spin structures, and excitation spectrum of coupled spin-1/2 trimer-chain for the Hamiltonian of Na_2_Cu_3_Ge_4_O_12_. **a** The level spectrum and corresponding wave functions of isolated trimer in presence of (left) only NN *J*_1_ and (middle) both NN and NNN interactions [*J*_1_, (*J*_3_ = *βJ*_1_ with *β* = 0.18)]. The first column shows the wave functions where isolated up and down spins are represented by up and down arrows, respectively and the singlet and triplet pairing between sites is denoted by lines and arrows, respectively. The corresponding total spin quantum number and magnetic quantum number are given by *S* and *M*, respectively. The last column shows the level spectrum for coupled trimers with *J*_2_ (=*αJ*_1_ with *α* = 0.18). The exchange interaction *J*_2_ introduces dispersion for each of the energy levels where the lowest level (A), middle level (B), and highest level (C) excitations are composed of dispersive spinons, doublons, and quartons, respectively. **b** Possible spin configurations of the ground state, spinon, doublon and quarton excitations. The red arrows are the flipped spins with respected to the ground state. Red dashed arrows show the delocalization of spins throughout the system. The greenish oval represents the trimers and the intertrimer interactions are shown by black dashed lines. The up and down spins are depicted by uparrow and downarrow and colour codes follow the text. **c** The dispersion relations and intensities [Dynamic spin structure factor *S*(*Q*,*w*)] for the spinon, doublon, and quarton excited states, over the full Brillouin zone along the chain, calculated by the DMRG (see ‘Methods’) for the Hamiltonian of Na_2_Cu_3_Ge_4_O_12_ (*J*_1_ = 235 K, *α* = 0.18, *β* = 0.18 and *g* = 2.06).

The schematic energy level spectrum and corresponding Eigen functions of coupled spin-1/2 trimers are shown in Fig. 2a. Na_2_Cu_3_Ge_4_O_12_ resembles weakly coupled trimers and its spectrum is mostly dominated by the isolated trimer. For an isolated spin-1/2 trimer (*α* = *γ* *=* 0) with the exchange interactions *J*_1_, the ground state of a trimer system is a doublet with two fold degenerate lowest energy *E*_0 _= –*J*_1_ for *β* = 0 and *E*_0 _= −0.955*J*_1_ for *β* = 0.18. The wave-function is a linear combination of one singlet and a free spin where singlet bond is always between the NN bonds (Fig. 2a). The doublet lowest excited state is also doubly degenerate and is at energy *E*_1 _= 0 for *β* = 0 and *E*_1_ = −0.135*J*_1_ for *β* = 0.18. Their wave function is a product of free spin with a singlet bond between NNN spins (Fig. 2a). The highest excitation is a quartet with total spin = 3/2; these energies are four-fold degenerate and are located at *E*_2_ = 0.5*J*_1_ for *β* = 0 and *E*_2_ = 0.545*J*_1_ for *β* = 0.18. The wave function is a linear combination of configurations which are direct product of a free spin and a triplet bond between two NN spins and product of a free spin and triplet bond between two NNN spins. All three spins in the same direction is another configuration for quartet (Fig. 2a). The weak antiferromagnetic exchange interaction between neighbouring trimers (smaller values of *α*) gives rise to a singlet ground state, since the lowest energy state of each trimer behaves as effective spin-1/2. A possible configuration of antiferromagnetically coupled trimers is shown in Fig. 2b. The lowest excitation energy modes are spinon modes generated by flipping one spin on trimer and it disperses throughout the system. One of the configurations of this excitation is shown in Fig. 2b. Higher energy excitation modes can be created by the flipping of a down spin in the initial configuration as well as a singlet bond between NN spins replaced by a singlet bond with NNN spins. This type of dispersing excitation is called a doublon (the terms doublon and quarton used here were introduced in ref. 26) where the change in total *S*^z^ is still one, as for the case in Fig. 2b. Another type of dispersive excitation is quarton where a single spin is flipped on a trimer to break the singlet bonds and polarize all three spins on a trimer or to convert the initial trimer configuration to a product of a free spin and a triplet bond between NNN (or NN) spins (Fig. 2b). The horizontal arrow in Fig. 2b represents the delocalization of an excitation. The doublon and quarton excitations, originating from internal trimer states, are almost localized for small values of *α*, and cannot be classified as standard magnons, or triplons, or spinons. For the larger values of *α*, the doublons and quartons lose their identity and fractionalize into the standard spinon continuum that emerges for *α* → 1, i.e. for the HAC. On the other hand, in the intermediate regime, doublon and quarton coexist with spinon-pair continua. The theoretical dispersion relations and the intensities of the spinon, doublon, and quarton excitations calculated by the hybrid ED/DMRG (see ‘Methods’) for *J*_1_ = 235 K, *α* = 0.18, *β* = 0.18 and *g* = 2.06 which correspond to Na_2_Cu_3_Ge_4_O_12_ are illustrated in Fig. 2c. Our dynamical results agree well with the results of several other numerical methods [ED^26^, ED with truncated Hilbert space^20^, Quantum Monte Carlo by applying a variant of the stochastic analytic continuation (QMC-SAC)^20^] (see Supplementary Note 6 for further details). The composite doublon, and quarton excitation states (marked as B and C, respectively) are gapped throughout reciprocal space, whereas, spinon-pair continua (marked as A) are gapless at specific wave vectors. All the three excitation modes have distinct energy ranges where the spinon-pair modes are found below 5 meV, while, the doublon, and quarton states appear at intermediate (*E* = *ħω* over 17–22 meV) and high (32–37 meV) energy ranges, respectively. The broadening of the doublon and quarton excitations appear due to their weak dispersion relations governed by the weaker *J*_2_ coupling (*α* = 0.18)^26^. For spinons, most of the intensity appears around the zone centre (*q* = π). The spectral weight of quarton modes are concentrated over *q* = (0.5–1.5)π. Whereas, spectral weight of doublon modes are distributed over *q* = (0.3–0.8)π and (1.2–1.7)π (Fig. 2c).

### Spin excitation

**Fig. 3:**
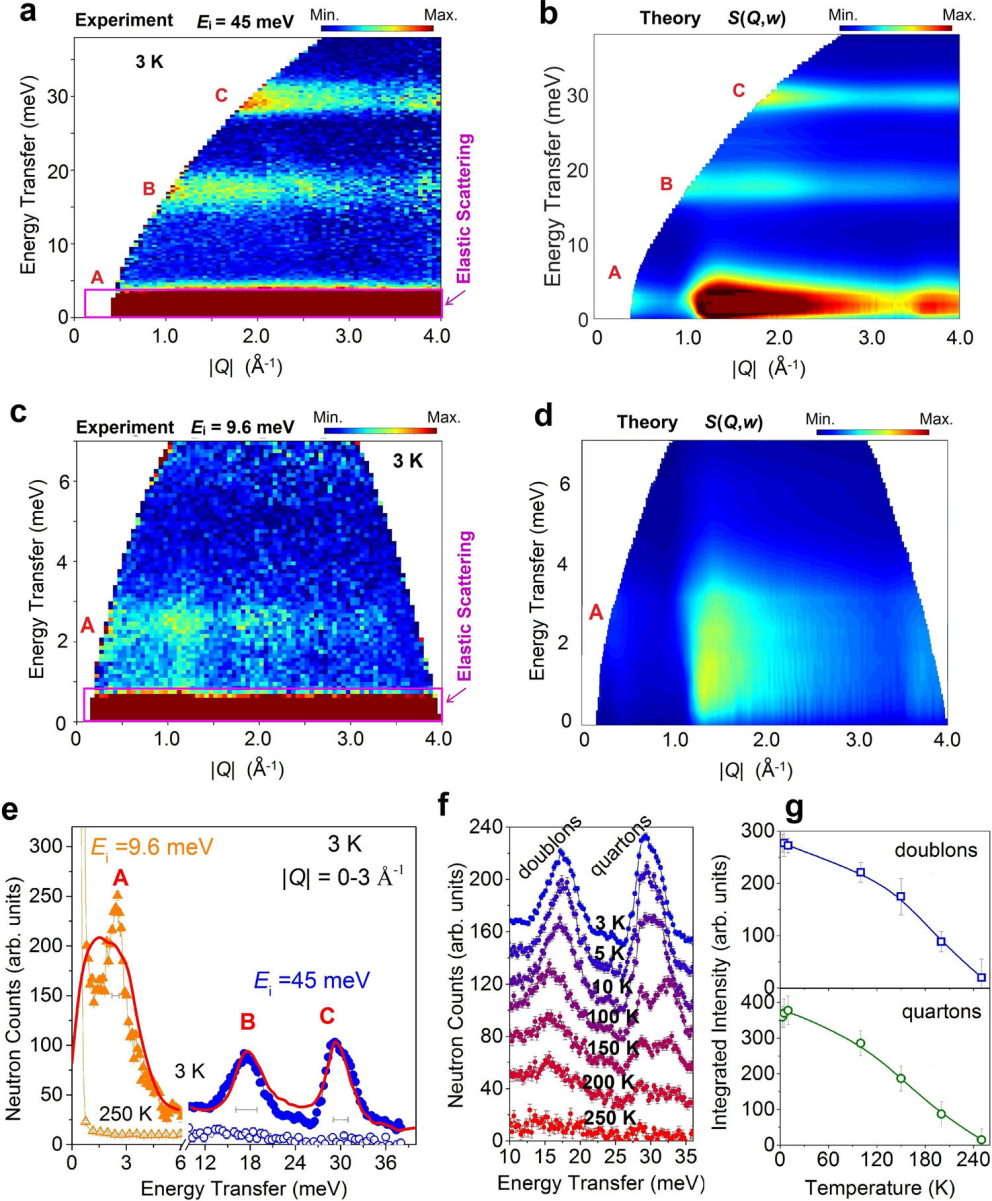
Dynamic structure factors of composite and spinon excitations in Na_2_Cu_3_Ge_4_O_12_ powders. **a**, **c** False-colour maps of the INS intensity measured at *T* = 3 K on the MERLIN spectrometer at ISIS facility, RAL, UK (in the 1D magnetic state above the *T*_N_ = 2.25 K) using a fixed incident energy of *E*_i_ = 45 and 9.6 meV, respectively (see ‘Methods’). The INS spectra have had an estimated phonon contribution subtracted (see ‘Methods’). The intensities are denoted by different colours, as indicated by the scales at each panel. The magenta boxes at the bottom of the each patterns represent the areas where the elastic scattering contributions are evident. **b**, **d** The excitation spectra for the spin-1/2 antiferromagnetic trimer-chain model for the Hamiltonian of Na_2_Cu_3_Ge_4_O_12_ (*J*_1_ = 235 K, *α* = 0.18, and *β* = 0.18) at *T* = 0, calculated using the hybrid ED/DMRG method. **e** Comparison of the scattering intensity with the theory. Energy cuts through the data (**a** and **c**) integrated over the |*Q*| range 0–3 Å^−1^. Error bars indicate the standard deviation assuming Poisson counting statistics. The instrumental resolution at the peak energies are shown by the horizontal bars. The red solid curves are the calculated intensities. **f** The temperature evolution of the doublons and quartons excitations which are found to be present up to a temperature ~ 250 K. **g** The temperature-dependent integrated intensity for the doublons and quartons excitations.

We performed INS experiments to measure the dynamical structure factors *S*(*Q*, *ω*) of Na_2_Cu_3_Ge_4_O_12_ (see ‘Methods’) in the 1D state at 3 K (*T*_N_ = 2 K). Figure 3a and c depicts the phonon background corrected magnetic excitation spectra of Na_2_Cu_3_Ge_4_O_12_ powders, measured with incident energies *E*_i_ = 45 and 9.6 meV, as a function of energy and wave vector which cover all the three spin excitation levels at ~3, 16, and 30 meV (A, B, and C). Although the directional-dependent information in the *S*(*Q*, *ω*) is lost due to the powder averaging, the powder *S*(|*Q*|, *ω*) preserves singularities arising in the density of states as a function of *E* = *ћω* and provides distinctive fingerprints of the Hamiltonian (1) which can be readily compared to theoretical calculations to estimate the *J* parameters. The higher energy modes B and C are gapped and weakly dispersive. Whereas, the low energy mode A is clearly dispersive in nature and shows a possible gapless minima at around wave vector transfer |*Q*| = 1.2 Å^−1^. The experimental spectra for Na_2_Cu_3_Ge_4_O_12_ are in good agreement with the powder-averaged DMRG results of DSF of a coupled trimer HAC system [Eq. (1)] with the parameters *J*_1_ = 235 K, *α* = 0.18, and *β* = 0.18 (Fig. 3b, d) in terms of the Eigen energies as well as spectral features. Such an agreement thus unambiguously provides an experimental realization of doublon and quarton excitations and their in-depth characterization. An energy cut through the data (integration of |*Q*| over 0–3 Å^−1^) (Fig. 3e) reveals all the three excitation modes as well as their respective intensity and spectral width. Comparisons with the theoretical DMRG results (shown by the red curves) have strong agreements overall, while the slight difference at low energies could be ascribed to the effects of 3D interchain couplings, which are weak and have not been considered in the calculations. Further, the temperature-dependent neutron scattering spectra (Fig. 3f, g) reveal that the doublon and quarton excitations persist up to ~250 K, which is within the energy range of the intra-trimer NN exchange constant *J*_1_ = 235 K, suggesting internal trimer excitations in the coupled trimer chain as their origin.

### Phase diagram

**Fig. 4:**
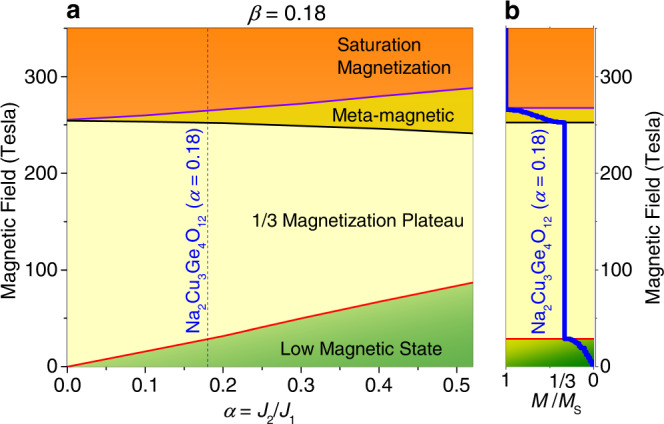
The magnetic phase diagram of Na_2_Cu_3_Ge_4_O_12_. **a** The evolution of the magnetic field induced states as a function of the inter-trimer exchange coupling strength *α* = *J*_2_/*J*_1_ (for *β* = 0.18). With the increasing *α* value, the 1/3 magnetization plateau state becomes narrower. The vertical dashed line represents the position of Na_2_Cu_3_Ge_4_O_12_. **b** The solid blue curve is the calculated magnetization curve for Na_2_Cu_3_Ge_4_O_12_ having *α* = 0.18 [Fig. 1d] illustrating the different magnetic states, i.e., low magnetic state for *M*/*M*_s_ < 1/3, 1/3 magnetization plateau for *M*/*M*_s_ = 1/3, meta-magnetic state for 1/3 < *M*/*M*_s_ < 1 and saturation magnetization for *M*/*M*_s_ = 1.

The effect of inter-trimer exchange coupling is illustrated by the simulated quantum phase diagram of the 1D trimer model [Eq. (1)] in the extended *α*-*H* space for *β* = 0.18 and *J*_1_ *=* 235 K [Fig. 4]. To explore the *α*-*H* space experimentally, the value of *α* can be tuned by a suitable chemical substitution or an application of pressure. For *α* = 0, the model is reduced to isolated trimers. In zero magnetic field, such isolated trimer system has discrete energy levels, with a doublet as the ground state. For an isolated trimer system, an application of an infinitesimal magnetic field leads to the onset of the 1/3 magnetization plateau state. The plateau phase exhibits the stability of the doublet state of the trimer. On increase in the applied magnetic field, the system shows a transition from the doublet state *M* = 1/2 (magnetization plateau state) to the quartet state *M* = 3/2 (the saturation magnetization state), and the magnetic field required for the transition is proportional to the plateau width *H*_c2_. The condition for doublet to quartet transition is ${E}_{0}-\frac{1}{2}g{\mu }_{B}{H}_{c2}={E}_{2}-\frac{3}{2}g{\mu }_{B}{H}_{c2}$ where *E*_0_ and *E*_2_ are the energy values for the doublet ground state and the quartet excited states in absence of magnetic field, respectively. The width of the 1/3 plateau is proportional to (*E*_2_ – *E*_0_) (=1.5*J*_1_). Now we turn our attention to an interacting trimer spin system as applicable for the present compound. In such a situation, any finite intertrimer interaction *J*_2_ induces a dispersive band for the doublet and quartet energy states. In this case also the 1/3 magnetization state gets stabilized, however, it requires a finite magnetic field value *H*_c1_ to induce an onset of the 1/3 magnetization plateau state. The *H*_c1_ increases with the increasing *J*_2_, whereas, the *H*_c2_ (the field at which the 1/3 magnetization plateau state ends) reduces with increasing *J*_2_. The width of plateau (*H*_c2_–*H*_c1_) thus decreases with increasing *J*_2_. The intertrimer coupling *J*_2_ also gives another threshold beyond the *H*_c2_ to reach the magnetization to its complete saturation. Such a state between the *H*_c2_ and the saturation magnetic field *H*_S_ is denoted as a meta-magnetic state [Fig. 4]. On the other hand, below the 1/3 plateau (i.e. *H* < *H*_c1_) all the magnetic states are denoted as low magnetic states [Fig. 4]. In the low magnetic states, the *M*–*H* curve varies linearly with *H* in the lower magnetization regime, but it has an algebraic variation as *M*(*H*)–*M*(*H*_c1_) ∝ (*H*−*H*_c1_)^1/2^ near the cusp of the plateau at *H*_c1_. The coefficient ½ of variation is reported near the cusp for various model quantum spin systems^27,28^. The phase diagram with the frustrated interaction *J*_3_ in the (*β*–*J*_1_) plane is shown in Supplementary Note 5.

Within the topologically protected quantum 1/3 magnetization-plateau state of the trimer chain, the nearest-neighbour spin-spin correlations and bipartite entanglement on three bonds (*S*_3i-2,3i-1_, *S*_3i-1,3i_ and *S*_3i,3i-1_) also exhibit interesting correlation^29^. Finite value of the bipartite quantum correlations in the trimer ground state, that persists up to a very high temperature (asymptotic limit of temperature *T* → ∞) has a potential role in quantum information processing^21^. Here the possible emergence of novel quasi-particles, by reconstruction of elementary excitations due to spontaneous symmetry breaking, is expected up to a temperature close to room temperature. Further, the quantum entanglement for Na_2_Cu_3_Ge_4_O_12_ reveals a very high decoherence (critical) temperatures of ~250 K (the quantum entanglement estimated following the Hilbert-Schmidt norm^18^ for *α* = 0 (see Supplementary Note 4, and Supplementary Fig. 5 for details). Such a high decoherence temperature, close to room temperature, is of special requirement for practical device applications.

In summary, the central finding of the present study is that Na_2_Cu_3_Ge_4_O_12_ is the first compound to realize the weakly coupled AFM trimer chain model and to reveal experimental observation of topological quantum excitations of novel quasi-particles doublon and quarton. The present work, thus, is expected to open up an avenue for the exploration of novel topological quantum states based on spin-trimers systems and beyond. The present work anticipates new directions of future theoretical and experimental studies to explore (i) high-energy fractionalization mechanism, (ii) synthesis of new materials or modifying the chemical composition to achieve low-field magnetization plateau state, as well as to vary the excitation energies and gaps of the newly observed topological quantum excitations, (iii) the evolution of the different types of excitations, especially when the doublons and quartons begin to form the conventional spinon continuum with the increasing inter-trimer exchange interaction (*α* = *J*_2_/*J*_1_), and (iv) the quantum topological excitation states at higher temperatures extending up to room temperature or beyond.

## Methods

### Sample preparation and characterization

Polycrystalline samples of Na_2_Cu_3_Ge_4_O_12_ were prepared through the solid state reaction method. High purity (>99.99%) reagents of Na_2_CO_3_, CuO and GeO_2_ were mixed together with a molar ratio 1:3:4. The mixture was annealed at 800 °C for total 40 h in air in a muffle furnace with several intermediate grindings. The phase purity of the powder samples was confirmed by Rietveld analysis of the room temperature X-ray diffraction pattern measured using a laboratory X-ray machine (see Supplementary Fig. 1).

### Neutron diffraction

Room temperature neutron powder diffraction pattern was measured on the powder diffractometer PD-II (*λ* = 1.2443 Å) at Dhruva reactor, Bhabha Atomic Research Centre, India (to derive crystal structural correlations) (see Supplementary Fig. 2).

### Dc-magnetization using PPMS

The temperature and field dependence of magnetization were measured using a commercial physical properties measurement system (PPMS) (Cryogenic Co. Ltd., UK). The dc-magnetization measurements were carried out over 1.5–300 K in the zero-field-cooled condition under 1 T of magnetic fields. Isothermal *M* vs *H* curve was measured at 3.0 K over the field range of ± 9 T (see Supplementary Note 2).

### High-pulse field magnetization

The high pulse-field magnetization measurements up to 60 T were performed at the Hochfeld Magnetlabor, HZDR, Dresden, Germany. The magnetization signal was detected by an induction method^30^ and corrected for the empty magnetometer background to obtain the sample magnetization.

### Inelastic neutron scattering

The inelastic neutron scattering (INS) measurements were performed on the high-flux neutron time-of-flight instruments MERLIN at the ISIS facility of the Rutherford Appleton Laboratory, Didcot, United Kingdom^31^. The INS spectra on MERLIN were recorded with a fixed incident neutron energy of *E*_i_ = 18 meV with the repetition rate multiplication (RRM) method^32,33^ by using a straight Gd Fermi chopper (speed was fixed to 250 Hz), which provides simultaneous measurements of INS patterns corresponding to incident energies of *E*_i_ = 45, 18, and 9.6 meV, respectively. About a 17-g powder sample was used for these INS measurements. The INS spectra were collected at several temperatures down to 3 K using a helium cryostat. The INS data were reduced using the MANTID software package^34^. The raw data were corrected for detector efficiency and time-independent background following standard procedures. The INS spectra are corrected for the background due to the phonon scatterings. The energy and wave vector-dependent phonon backgrounds are estimated from the measured spectra at 300 K with an application of a required Bose factor.

### High-field ESR

The electron spin resonance (ESR) measurements were performed employing a 16 T transmission-type ESR spectrometer^35^. Measurements were done in a frequency of 136 GHz at 7.3 and 18.7 K, using a VDI microwave-chain radiation source (product of Virginia Diodes, Inc., USA). An InSb hot-electron bolometer (QMC Instruments Ltd., UK) was used to record the spectra.

### Numerical calculations

The temperature dependence of the thermodynamic quantities for Na_2_Cu_3_Ge_4_O_12_ were calculated using the hybrid exact diagonalization (ED) and density matrix renormalization group (DMRG) method (hybrid ED/DMRG)^36,37^. It combines exact diagonalization (ED) of short chains with density matrix renormalization group (DMRG) calculations of progressively longer chains. The hybrid ED/DMRG technique has been extensively applied to 1D spin systems^36^ and fermionic systems^37^. The main advantage of this method is that the full spectrum of large systems of N spins is not needed. Since thermal fluctuations limit the range of spin correlations, small systems using ED provides the thermodynamic limit at high temperature where the system size exceeds the correlation length. However, the study of thermodynamics at low temperature requires systems of large number of spins to reach the thermodynamic limit due to presence of large correlation length. The partition function is the sum of the Boltzmann probability of all energy states, and has a significant contribution from low energy states at low temperature. The higher excited states have exponentially small contributions, therefore, the accurate low-temperature properties require only accurate low-lying energy states. The DMRG method is well known for its accurate calculation of low-lying states and this property of DMRG is exploited to calculate the low-temperature properties.

In the hybrid ED/DMRG method density matrix of the system block is calculated using the projection of all low lying energy states of the superblock and the details of projection procedure is explained in ref. 36. For the trimer model in Eq. 1, we have used ED to calculate the thermodynamics at high temperature and progressively larger system size using DMRG yields low energy excitations and extends the thermodynamics to lower temperature. We have retained up to 700 eigenstates in the density matrix for the DMRG calculation and calculated 400 states in each *S*^Z^ sector. It gives access to the accurate temperature dependence down to *T* ~ 0.01 *J*_1_. The Eq. (1) was used with different sets of values of *J*_1_, *α*, and *β* to reproduce the experimentally observed *χ*(*T*) and *M*(*H*).

The dynamical spin structure factor for Na_2_Cu_3_Ge_4_O_12_ was calculated using the DMRG method along with the correction vector method^38–40^ for *N* = 48 spins i.e.,16 trimers (Figs. 2c and 3b). To compare the calculated spinon excitation spectrum with the high-resolution experimental INS spectrum with *E*_i_ = 9.6 meV (Fig. 3c), the low energy spinon spectrum is also calculated for *N* = 96 spins i.e., 32 trimers (Fig. 3d) which minimizes the finite size effect. The dynamical structure factor is defined as

2 $$S\left(q,\omega \right)=\mathop{\sum}\limits_{n}\frac{{\left|\left\langle {\psi }_{n}\left|{S}_{q}^{\alpha }\right|{\psi }_{0}\right\rangle \right|}^{2}}{{E}_{n}-\left({E}_{0}+\omega \right)+i\eta }$$

Here *E*_0_ and *E*_n_ are the energies of the ground state and *n*th excited state, respectively. The *ω* and *q* represent the energy and momentum, respectively, transferred to the lattice. The *η* is the broadening factor. $|{\psi }_{0}\rangle$ is the ground state wave function and $|{\psi }_{n}\rangle$ is the *n*th excited state wave-function. If a denotes the *x*, *y*, and *z* component of spin, we can define

3 $${S}_{q}^{\alpha }=\sqrt{\frac{2\pi }{N}}\mathop{\sum}\limits_{i}{e}^{{iqj}}{S}_{j}^{\alpha }$$

## Supplementary information

Supplementary Information

## Data Availability

The datasets for the inelastic neutron scattering experiment on the time-of-flight MERLIN spectrometer are available from the ISIS facility, Rutherford Appleton Laboratory data portal (DOI: 10.5286/ISIS.E.RB1910432). All other data of this work are available with the authors on request.

## Code availability

The code is available upon reasonable request from M.K.
