# Supplementary material for: Emergent many-body composite excitations of interacting spin-1/2 trimers
Source: Nat Commun. 2022 Nov 12;13:6888. doi: 10.1038/s41467-022-34342-1 (PMC9653475; doi:10.1038/s41467-022-34342-1)
Supplement: Supplementary file 1 — Supplementary Information [file 41467_2022_34342_MOESM1_ESM.pdf]

Supplementary informations for

Emergent many-body composite excitations of interacting spin-1/2 trimers

Anup Kumar Bera[1,2], S. M. Yusuf[1,2*], Sudip Kumar Saha[3], Manoranjan Kumar[3], David Voneshen[4,5], Yurii Skourski[6] and Sergei A. Zvyagin[6]

[1]Solid State Physics Division, Bhabha Atomic Research Centre, Mumbai 40085, India
[2]Homi Bhabha National Institute, Anushaktinagar, Mumbai 400094, India
[3]S. N. Bose National Centre for Basic Sciences, Block JD, Sector III, Salt Lake, Kolkata 700106, India
[4]ISIS Facility, STFC Rutherford Appleton Laboratory, Harwell Oxford, Didcot OX11 0QX, UK
[5]Department of Physics, Royal Holloway University of London, Egham, TW20 0EX, UK
[6]Dresden High Magnetic Field Laboratory (HLD-EMFL), Helmholtz-Zentrum Dresden-Rossendorf, 01328 Dresden, Germany

e-mail: smyusuf@barc.gov.in

Inventory of Supporting Information

**Supplementary Note 1: Crystal Structure:** Supplementary Figs. 1 and 2 Supplementary Tables 1, 2 and 3	**Page 2**
**Supplementary Note 2: Bulk magnetic properties:** Supplementary Fig. 3	**Page 7**
**Supplementary Note 3: Electron Spin Resonance (ESR) spectroscopy:** Supplementary Fig. 4	**Page 8**
**Supplementary Note 4: Quantum Entanglement Parameter:** Supplementary Fig. 5	**Page 9**
**Supplementary Note 5: Phase diagram with the frustrated interaction J_3 (βJ_1):** Supplementary Fig. 6	**Page 11**
**Supplementary Note 6: Comparison of simulations of neutron structure factors:** Supplementary Fig. 7	**Page 12**
**Supplementary References:**	**Page 14**

Supplementary Note 1: Crystal Structure

The crystal structure of $Na_2Cu_3Ge_4O_{12}$ has been investigated by the combined analysis of x-ray and neutron diffraction patterns at room temperature. The Rietveld analysis of the diffraction patterns (Supplementary Fig. 1) reveals that the compound crystallizes in the triclinic symmetry with group *P*-1 in agreement with that reported earlier. The lattice parameters are determined to be a = 6.1824(3) Å, b=7.6912(3) Å, c = 5.4721(2) Å, α= 102.388(2)°, β = 93.074(3)°, and γ= 87.579(3)° with unit cell volume V = 253.65(2). The refined atomic positions, isotropic thermal parameters, and site occupation numbers are given in Supplementary Table-1.

In the present crystal structure, the magnetic Cu^{2+} ions are distributed at two Wyckoff sites [Cu1(1*a*), and Cu2 (2*i*)]. The Na ions have single, Ge ions have two and O ions have six Wyckoff positions. The most prominent crystal structural feature of $Na_2Cu_3Ge_4O_{12}$ is the periodic arrays of Cu_3O_8 trimers formed by edge-sharing three CuO_4 square planes in a linear fashion with the intra-trimer copper distances of 3.048(5) Å, which is indicated by the nearly 180° dihedral angles. Within a given trimer, the central Cu1 atom is coordinated nearly square planar [coordination partners: 2 × O1 [Cu1-O1=1.915(6) Å] and 2 × O4 [Cu1-O4=2.049(7)]] and the two terminal Cu2 atoms are coordinated distorted square planar [coordination partners: O1 [2.001(9) Å] and O4 [1.957(7) Å], O5 [1.881(9) Å], and O6 [1.918(8) Å]]. The bridging angles between the square planar are Cu1–O1–Cu2=102.2(4) and Cu1–O4–Cu2= 99.0(4) (Supplementary Table-2). These trimers are embedded in an extended lattice, connected by corner sharing GeO_4 tetrahedra, exhibiting a pseudo-one-dimensional channel framework. The electro positive Na^+ cations reside in channels constructed by six corner-sharing polyhedral units (2 × CuO_4 and 4 ×GeO_4), as shown in Supplementary Fig. 2. In an isolated trimer, three Cu ions are coupled by intratrimer exchange interactions (J_1). Such trimers are coupled in a zigzag chain by intertrimer exchange interaction J_2 along the chain. Within a given trimer, the two edged Cu^{2+} (Cu2) ions are coupled by the second nearest neighbour exchange interaction J_3. The details of the superexchange pathways are given in Supplementary Table-3. The Cu_3O_8 trimers chains are well separated by the nonmagnetic Na^+ and Ge^{4+} ions resulting in a weak interchain interaction J_{int}.

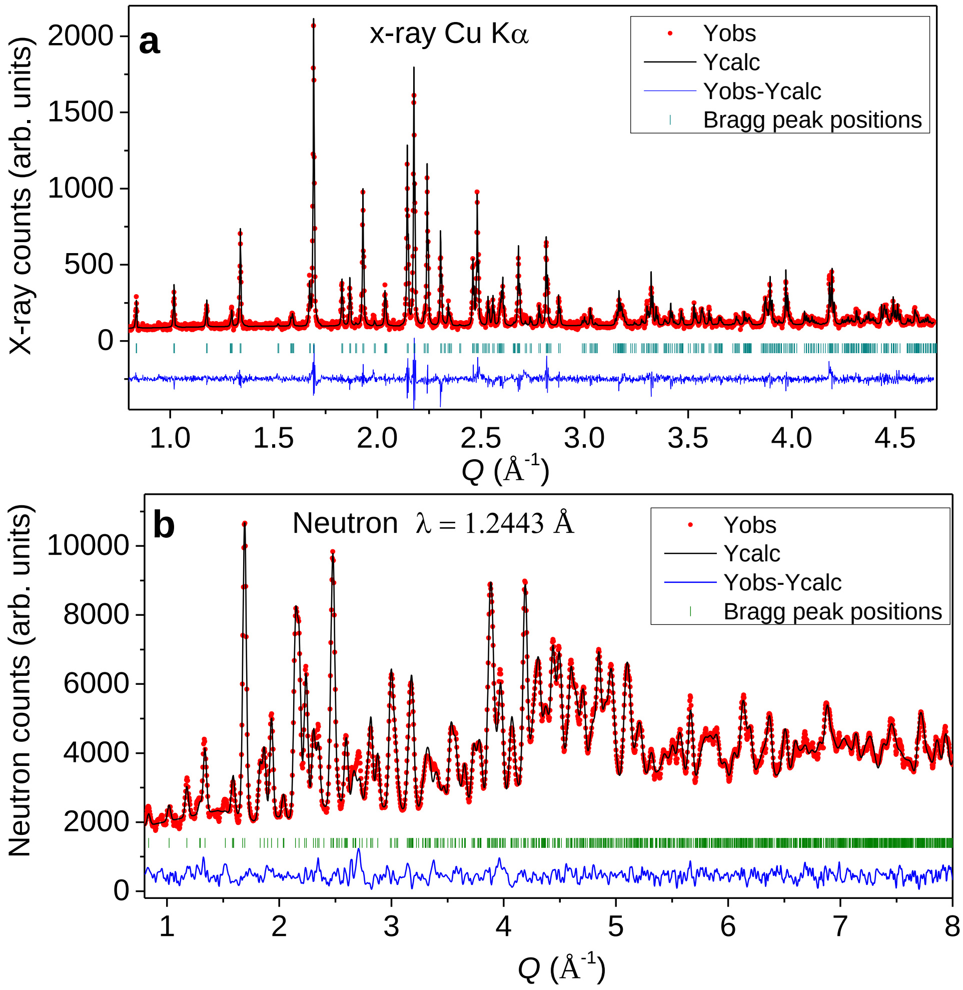

Supplementary Fig. 1. **a** and **b** The x-ray and neutron diffraction patterns, respectively, of $Na_2Cu_3Ge_4O_{12}$ at room temperature. Solid circles represent the experimental data points and the black line (through the data points) is calculated pattern by Reitveld method with the space group *P*-1.

Supplementary Table 1. The Rietveld refined atomic positions, isotropic thermal parameters, and site occupation numbers for $Na_2Cu_3Ge_4O_{12}$ at room temperature. Space group: *P*-1, *a* = 6.1824(3), *b*=7.6912(3) , *c* = 5.4721(2), α= 102.388(2), β = 93.074(3), and γ= 87.579(3).

Atom	Site	*x*/*a*	*y*/*b*	*z*/*c*	$10^2\times B_{iso}$ (Å^2)	*Occ.*
Na	2*i*	0.2494(12)	0.5955(13)	0.9174(13)	1.34(2)	1.0
Cu1	1*a*	0	0	0	0.87(2)	1.0
Cu2	2*i*	0.1632(14)	0.8231(13)	0.4873(12)	0.70(1)	1.0
Ge1	2*i*	0.2209(7)	0.3676(6)	0.3067(8)	0.57(3)	1.0
Ge2	2*i*	0.39881(8)	0.18278(6)	0.76148(9)	0.64(2)	1.0
O1	2*i*	0.0053(9)	0.2246(8)	0.2331(12)	0.93(1)	1.0
O2	2*i*	0.4021(9)	0.3184(8)	0.0644(11)	1.04(3)	1.0
O3	2*i*	0.3646(7)	0.3493(7)	0.5772(12)	1.07(8)	1.0
O4	2*i*	0.1749(11)	0.0550(9)	0.7197(12)	1.20(3)	1.0
O5	2*i*	0.6580(13)	0.0965(7)	0.7305(11)	1.01(3)	1.0
O6	2*i*	0.1273(9)	0.5843(8)	0.2996(10)	1.05(4)	1.0

Supplementary Table 2. The local crystal structural parameters for $Na_2Cu_3Ge_4O_{12}$; bond lengths, and bond angles at room temperature.

Site	bond length (Å)		bond angle (◦)	
Cu1	Cu1–O1	1.915(6) 2×	O1–Cu1–O1	180.0(7)
	–O4	2.049(7) 2×	O1–Cu1–O4	100.7(5) 2×
				79.3(5) 2×
			O4–Cu1–O4	180.0(7)
Cu2	Cu2–O1	2.001(9)	O1-Cu2-O4	79.5(5)
	–O4	1.957(7)	O1-Cu2-O5	169.3(7)
	–O5	1.881(9)	O1-Cu2-O6	91.5(5)
	–O6	1.918(8)	O4-Cu2-O5	90.5(5)
			O4-Cu2-O6	171.0(6)
Ge1	Ge1–O1	1.740(8)	O1–Ge1–O2	109.0(6)
	–O2	1.750(8)	O1–Ge1–O2	113.7(6)

	–O3	1.714(8)	O1–Ge1–O6	109.0(6)
	–O6	1.749(8)	O2–Ge1–O3	107.1(6)
			O2–Ge1–O6	103.4(6)
			O3–Ge1–O6	114.1(6)
Ge2	Ge2–O2	1.759(7)	O2–Ge1–O3	100.2(6)
	–O3	1.792(9)	O2–Ge1–O4	108.8(6)
	–O4	1.708(9)	O2–Ge1–O5	104.2(6)
	–O5	1.711(8)	O3–Ge1–O4	109.7(7)
			O3–Ge1–O5	107.8(6)
			O4–Ge1–O5	123.5(7)

Supplementary Table 3. Possible pathways for the exchange interactions J_1, J_2 and J_3, respectively. The Cu...Cu direct distances, metal oxide (M–O) bond lengths and bond-angles for the exchange interactions J_1, J_2 and J_3 in $Na_2Cu_3Ge_4O_{12}$ at room temperature.

**Exchange interaction**	**Pathways**	**Cu...Cu direct distance (˚A)**	**Bond lengths (Å)**	**Bond angles (deg.)**
J_1	Cu1–O–Cu2	Cu1–Cu2= 3.048(5)	Cu1–O1= 1.915(6)<br>Cu2–O1= 2.001(9)	Cu1–O1–Cu2=102.2(4)
			Cu1–O4= 2.049(8)<br>Cu2–O4= 1.957(8)	Cu1–O4–Cu2= 99.0(4)
J_2	Cu1–O1–Ge1–O6–Cu2 (along b axis)	Cu1–Cu2= 6.420(5)	Cu1–O1= 1.915(6)<br>Ge1–O1=1.740(8)<br>Ge1–O6=1.749(9)<br>Cu2–O6= 1.918(8)	Cu1–O1–Ge1=127.2(4)<br>O1–Ge1–O6=109.0(4)<br>Ge1–O6–Cu2=139.3(5)
J_3	Cu2–O1–O4–Cu2	Cu2–Cu2= 6.096(10)	Cu2–O1= 2.001(9)<br>O1–O4=3.053(9)<br>Cu2–O4= 1.957(8)	Cu2–O1–O4=143.3(4)<br>O1–O4–Cu2=137.16(4)
	Cu2–O1–Cu1–O4–Cu2		Cu2–O1= 2.001(9)<br>Cu1–O1=3.053(9)<br>Cu1–O4= 1.957(8)<br>Cu2–O4= 1.957(8)	Cu1–O1–Cu2=102.2(4)<br>Cu1–O4–Cu2= 99.0(4)

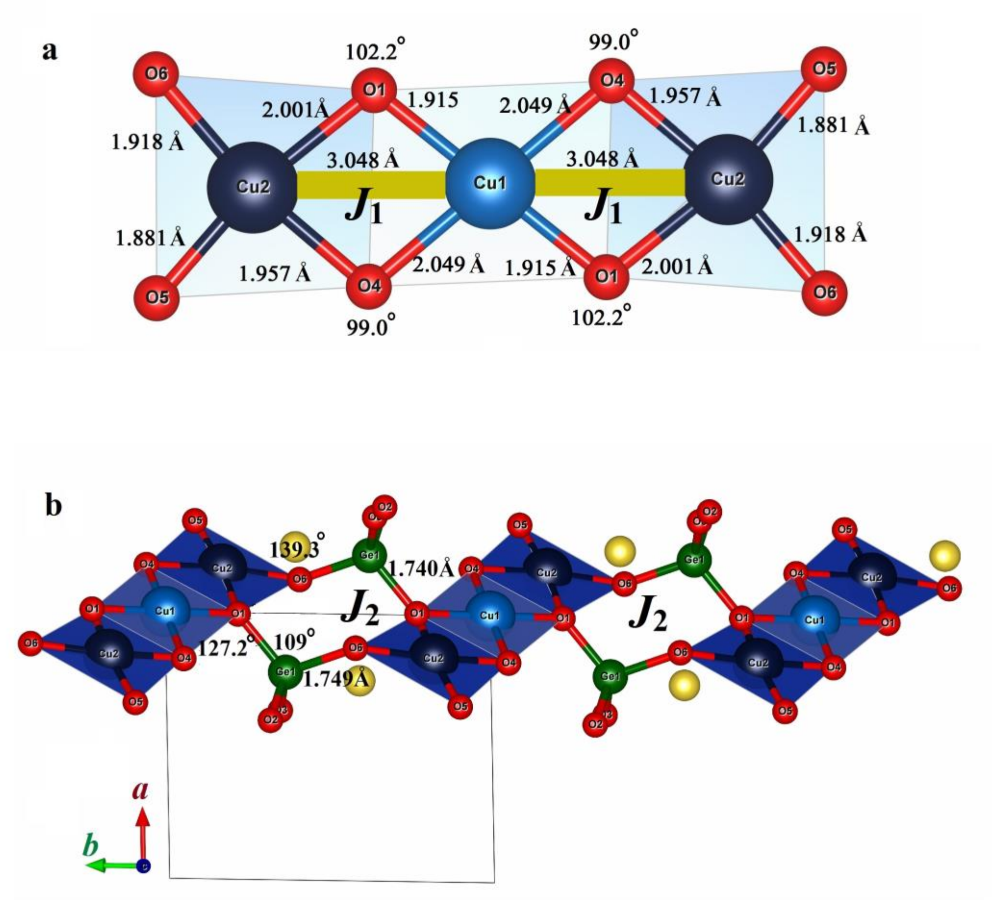

Supplementary Fig. 2. a The local crystal structure of an individual trimer unit of $Na_2Cu_3Ge_4O_{12}$. The experimentally measured values of the bond lengths, bond angles and the Cu-Cu distances at room temperature are shown. **b** The intertrimer connections along the crystallographic *b* axis through the Ge_1O_4 tetrahedra.

Supplementary Note 2: Bulk magnetic properties:

The temperature dependent susceptibility (χ vs T) curve measured under a magnetic field of B = 1 T is shown in Supplementary Fig. 3a. The $\chi(T)$ curve shows a broad maximum at T_{max} ~ 11 K due to the growth of the antiferromagnetic short-range order with decreasing T. With the further lowering of temperature, $\chi(T)$ curve shows an anomaly at T ~ 2 K (the inset of Supplementary Fig. 3a) revealing a magnetic long-range antiferromagnetic ordering in $Na_2Cu_3Ge_4O_{12}$ at T_N~ 2 K. The magnetic long-range ordering below T_N~ 2 K was reported by temperature dependent specific heat, nuclear magnetic resonance (NMR) and capacitance studies[1,2]. The χT vs T curve (Supplementary Fig. 3b) below ~ 300 K reveals a deviation from a constant value suggesting the deviation from the paramagnetic behaviour.

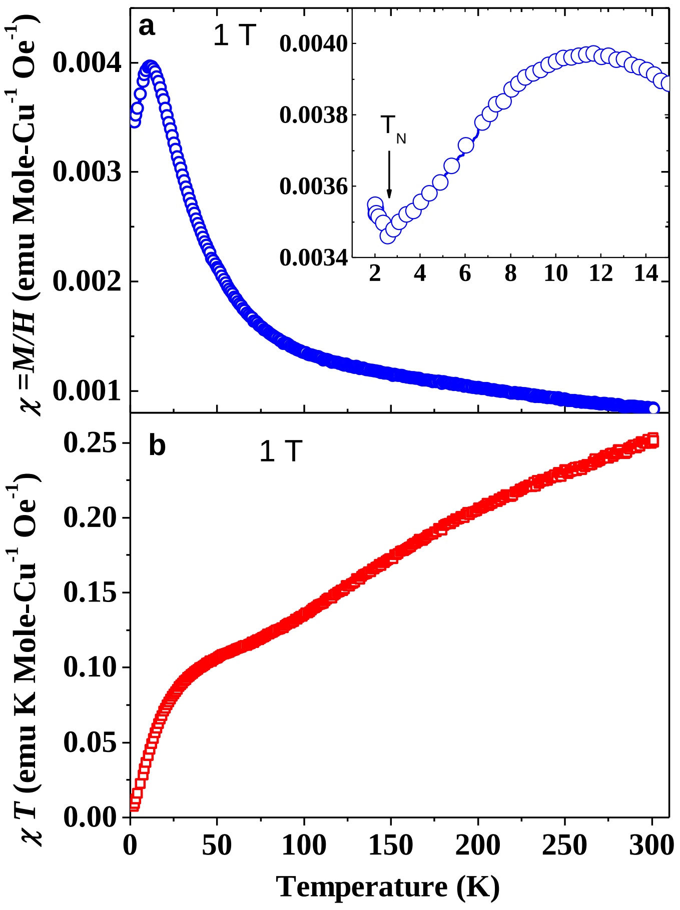

Supplementary Fig. 3. a The temperature dependent susceptibility [$\chi(T)$] curve measured on powder sample under a magnetic field B= 1 T. Inset highlights the low temperature region where a kink appears at the T_N =2 K. **b** The χT vs. T curve revealing the deviation from the a constant value as expected for a paramagnetic state having Curie behaviour and indicates the presence of magnetic correlations up to about room temperature as compared to low magnetic ordering temperature T_N ~ 2. K.

Supplementary Note 3: Electron Spin Resonance (ESR) spectroscopy:

The ESR powder spectra measured at a frequency of 104 GHz in the 2 – 70 K temperature range are shown in Supplementary Fig. 4. The spectral weight has been found distributed approximately between 3.13 and 3.6 T, which for this frequency corresponds to the *g*-factors ranging from 2.36 to 2.06, respectively. The spectra exhibit a tiny but noticeable broadening upon approaching the transition into the magnetically ordered state at T_N =2 K (compare, e.g., the spectra at 10 and 2 K).

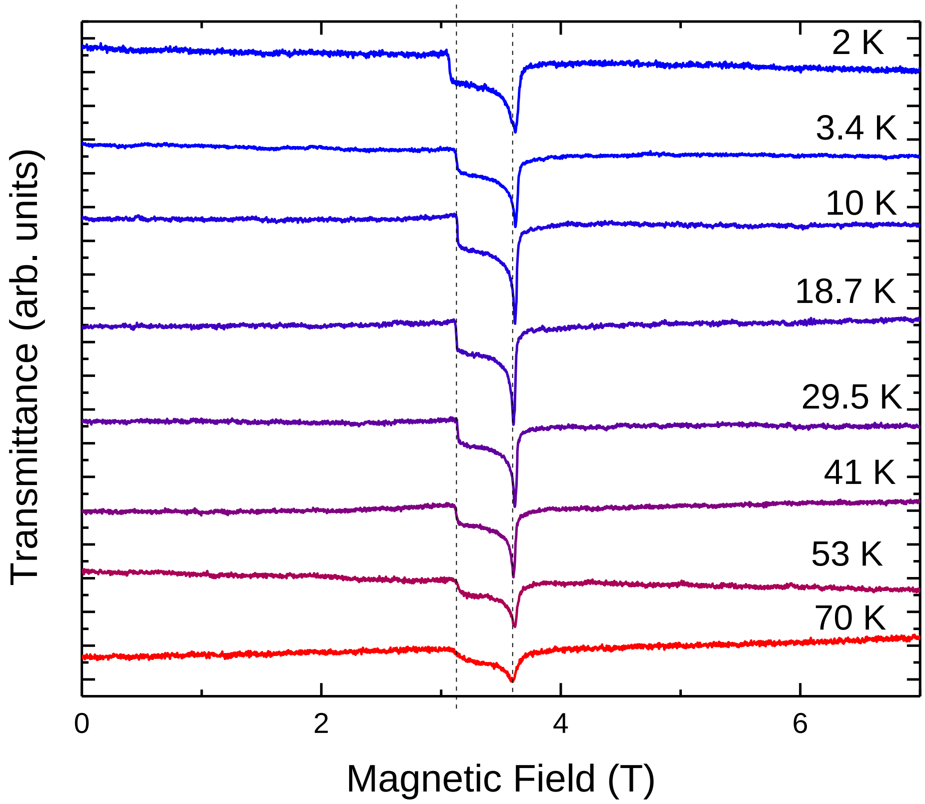

Supplementary Fig. 4. Examples of ESR spectra in $Na_2Cu_3Ge_4O_{12}$, measured at a frequency of 104 GHz at different tempertaures (the spectra are offset for clartity). The dash lines correspond to the *g*-factors 2.36 and 2.06.

Supplementary Note 4: Quantum Entanglement Parameter:

Quantum entanglement is a physical phenomenon that occurs when a group of particles interact among each other such a way that the quantum state of individual particle cannot be described independently of the state of the others, including when the particles are separated by a large distance. Entanglement is a primary feature of quantum mechanics lacking in classical mechanics. Quantum entanglement, the most prominent example of quantum correlations, serves as the fundamental resource in several quantum information processing tasks such as quantum computation and communication[3]. To measure quantum entanglement as a physical quantity, by using the Hilbert-Schmidt norm[4], Del Cima *et al.*[5], formulated a general method for spin-1/2 antiferromagnetic trimer. The weak inter trimer exchange interaction ($\alpha << 1$) are neglected for simplicity. The quantum entanglement measure $E(J,T)$ as a function of the temperature (T) and the exchange coupling (J) is defined as

$$E(J,T) = E_0 max\left[0, \left(2\left|\frac{3}{8}\frac{1+e^{\frac{J}{k_BT}}+10e^{\frac{3J}{2k_BT}}}{1+e^{\frac{J}{k_BT}}+2e^{\frac{3J}{2k_BT}}} - 1\right| + \frac{1}{2} - \frac{3}{8}\frac{1+e^{\frac{J}{k_BT}}+10e^{\frac{3J}{2k_BT}}}{1+e^{\frac{J}{k_BT}}+2e^{\frac{3J}{2k_BT}}}\right)\right] \quad (1)$$

with $\lim_{T\to 0} E(J,T) = \frac{11}{8}E_0 = \frac{11}{32}$

The entanglement critical temperature (T_c) is defined as the temperature where the quantum entanglement measure, $E(J,T)|_{T=T_c} \equiv 0$ vanishes. Thus, for temperatures below the critical (decoherence) temperature ($T < T_c$), the system is entangled, whereas, for temperatures above it, $T > T_c$, the system experiences quantum decoherence, it assumes a separable state. We have estimated the quantum entanglement measure $E(J,T)$ for $Na_2Cu_3Ge_4O_{12}$ following the above equation. The temperature dependence of the $E(J,T)$ is shown in Supplementary Fig. 5 which reveal a very high T_c of ~ 310 K as the exchange coupling protects the system from decoherence as temperature increases. Such a high decoherence temperature makes $Na_2Cu_3Ge_4O_{12}$ as a suitable candidate for practical device applications.

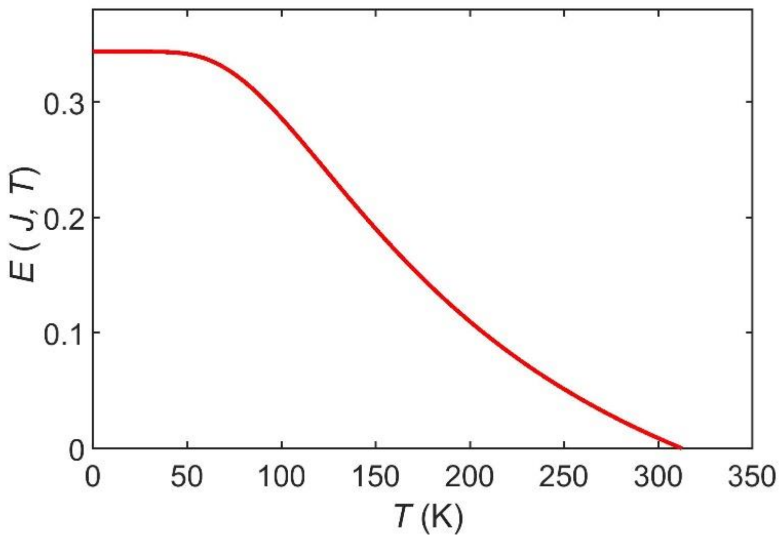

Supplementary Fig. 5. The quantum entanglement measure, $E(J,T)$, for $Na_2Cu_3Ge_4O_{12}$, with J/k_B = −235 K.

Supplementary Note 5: Phase diagram with the frustrated interaction J_3 (βJ_1):

The phase diagram of trimer model in Eq. 1 in β-H parameter space is shown for α=0 and 0.18 at J_1 = 235 K in Supplementary Figs. 6a and 6b, respectively. For α = 0 and finite β, the system behaves as isolated triangles with β as exchange interaction at the base of the triangle. The ground state is a doublet M = 1/3 state and has a finite gap which corresponds to gap between doublet and quartet states, therefore it requires large field to achieve the higher magnetization as shown in Supplementary Fig.6a. For α = 0.18 and $\beta \leq$ 1.5, each trimer has effective spin 1/2 which are coupled antiferromagnetically and forms a singlet ground state. It requires finite field to reach the M = 1/3 plateau phase and at higher values of H the system accesses metamagnetic phase and saturated magnetization. Large β (~1 or >1) favours the weakly coupled triangles configuration where base of each triangle has strong AFM coupling forming a strong dimer singlet. For 1.1 <β < 1.5, spins at the apex of a triangle are coupled effectively with weak AFM exchange and the ground state is still in the singlet state and it requires small H to achieve M = 1/3 plateau state. For $\beta \geq$ 1.5, the apex spins are coupled ferromagnetically resulting M = 1/3 plateau state as a ground state and this state has large energy gap. At β ~ 1 the width of the metamagnetic state increases due to formation of strong singlet dimers at the base of the triangles and it is proportional to the energy required to break the singlet dimers.

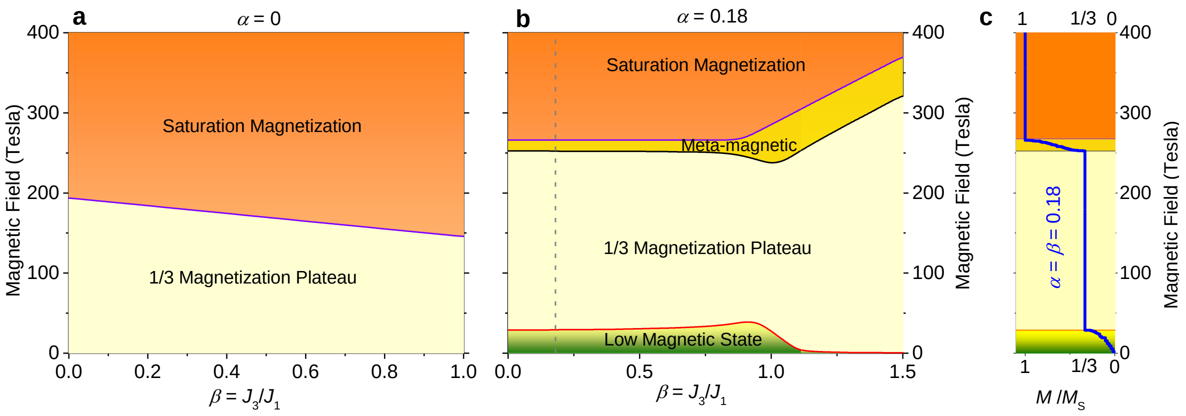

Supplementary Fig. 6. The quantum phase diagram of the trimer model in Eq. 1 in β-H parameter space at J_1 = 235 K for **a** α=0 and **b** α=0.18. **c** the definition of the magnetic states with a representative magnetization curve for $Na_2Cu_3Ge_4O_{12}$ (α=β=0.18), i.e., low magnetic state for M/M_s < 1/3, 1/3 magnetization plateau for M/M_s = 1/3, meta-magnetic state for 1/3 < M/M_s < 1 and saturation magnetization for M/M_s =1.

Supplementary Note 6: Comparison of simulations of neutron structure factors:

The DMRG with the correction vector method is a well-established numerical technique to calculate the dynamical structure factor $S(q,\omega)$ [6-9] and other dynamical properties [10,11] for various model 1D spin systems. To compare the DMRG calculated $S(q,\omega)$ with the results derived from all the three calculation methods (ED, ED with truncated Hilbert space, QMC-SAC calculations) reported in Refs. [12,13], we use N=48 spins with broadening factor (η=0.07). We calculate $S(q,\omega)$ at α=0.2 and β=0 and compare the results for g=0.2 in Refs. [12,13], i.e, (i) ED with truncated Hilbert space with N = 24 spins (Fig. 8b of Ref. 12), (ii) QMC-SAC calculation with N = 192 spins, and η=0.05 (Fig 2b of Ref. 12) and, (iii) ED for N=30 spins [Fig. 2(b) of Ref. 13]. It is evident that the calculated $S(q,\omega)$ in the present study (Supplementary Fig. 7) revealing the signatures of spinon, doublon and quarton excitation modes are in good agreement with the reported values (Refs. 12,13). The energy modes of the doublon and quarton match well with the calculated results using all the three methods. For the spinon modes, the lower energy region matches well with all the three methods, whereas, the higher energy region matches better with the ED calculation. The QMC-SAC results may be reproduced using a larger system size in the present calculation involving the DMRG with correction vector method.

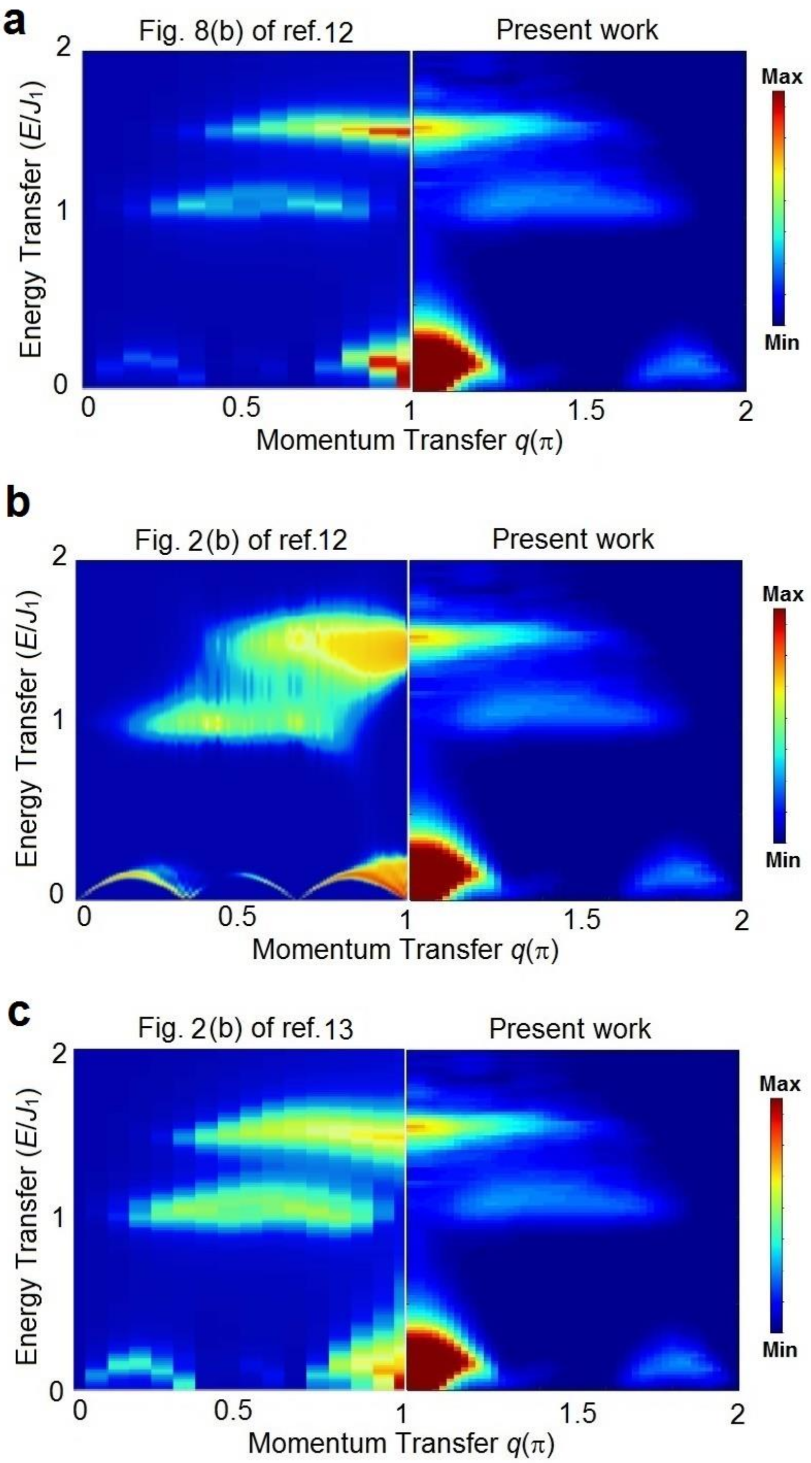

Supplementary Fig. 7. Comparison of the calculated dynamic structure factors calculated by **a** ED with truncated Hilbert space [Fig. 8(b) of Ref. 12] and **b** Quantum Monte Carlo (QMC) by applying a variant of the stochastic analytic continuation [SAC) [Fig. 2(b) of Ref. 12] and **c** ED [Fig 2(b) of Ref. 13] methods with the present calculation by ED/DMRG method.

Supplementary References:

1 Yasui, Y., Kawamura, Y., Kobayashi, Y. & Sato, M. Magnetic and dielectric properties of one-dimensional array of S = 1/2 linear trimer system $Na_2Cu_3Ge_4O_{12}$. *Journal of Applied Physics* **115**, 17E125, (2014).

2 Mo, X., Etheredg, K. M. S., Hwu, S.-J. & Huang, Q. New Cuprates Featuring Ladderlike Periodic Arrays of $[Cu_3O_8]_{10}^-$Trimeric Magnetic Nanostructures. *Inorganic Chemistry* **45**, 3478, (2006).

3 Silva, S. L. L. d. Entanglement of spin-1/2 Heisenberg antiferromagnetic quantum spin chains. *Quantum Stud.:Math. Found.*, (2017).

4 Dahl, G., Leinaas, J. M., Myrheim, J. & Ovrum, E. A tensor product matrix approximation problem in quantum physics. *Linear Algebra and its Applications* **420**, 711-725, (2007).

5 Cima, O. M. D., Franco, D. H. T. & Silva, S. L. L. d. Quantum entanglement in trimer spin-1/2 Heisenberg chains with antiferromagnetic coupling. *Quantum Studies: Mathematics and Foundations* **3**, 57-63, (2016).

6 Dutton, S. E., Kumar, M., Mourigal, M., Soos, Z. G., Wen, J. J., Broholm, C. L., Andersen, N. H., Huang, Q., Zbiri, M., Toft-Petersen, R. & Cava, R. J. Quantum Spin Liquid in Frustrated One-Dimensional ${\mathrm{LiCuSbO}}_{4}$. *Physical Review Letters* **108**, 187206, (2012).

7 Parvej, A. & Kumar, M. Multipolar phase in frustrated spin-1/2 and spin-1 chains. *Physical Review B* **96**, 054413, (2017).

8 Pereira, R. G., Sirker, J., Caux, J. S., Hagemans, R., Maillet, J. M., White, S. R. & Affleck, I. Dynamical Spin Structure Factor for the Anisotropic Spin-$1/2$ Heisenberg Chain. *Physical Review Letters* **96**, 257202, (2006).

9 Onishi, H. Magnetic Excitations of Spin Nematic State in Frustrated Ferromagnetic Chain. *J. Phys. Soc. Jpn.* **84**, 083702 (2015).

10 Ramasesha, S., Shuai, Z. & Brèdas, J. L. Correction vector method for exact dynamic NLO coefficients in restricted configuration space,. *Chem. Phys. Lett.* **245**, 224, (1995).

11 Jeckelmann, E. Dynamical density-matrix renormalization-group method. *Phys. Rev. B* **66**, 045114, (2002).

12 Cheng, J.-Q., Li, J., Xiong, Z., Wu, H.-Q., Sandvik, A. W. & Yao, D.-X. Fractional and composite excitations of antiferromagnetic quantum spin trimer chains. *npj Quantum Materials* **7**, 3, (2022).

13 Cheng, J.-Q., Li, J., Xiong, Z., Wu, H.-Q., Sandvik, A. W. & Yao, D.-X. Fractional and composite excitations of antiferromagnetic quantum spin trimer chains. *arXiv:2011.02448v2*, (2021).
